# Carbon and Calcium Carbonate Export Driven by Appendicularian Faecal Pellets in the Humboldt Current System off Chile

**DOI:** 10.1038/s41598-019-52469-y

**Published:** 2019-11-11

**Authors:** A. Eduardo Menschel, Humberto E. González

**Affiliations:** 1 Programa Doctorado en Oceanografía de la Universidad de Concepción and Instituto de Ciencias Marinas y Limnológicas, Universidad Austral de Chile, Research Centre on Dynamics of High Latitude Marine Ecosystems (FONDAP-IDEAL), Casilla, 567 Valdivia Chile; 2 Instituto de Ciencias Marinas y Limnológicas, Universidad Austral de Chile, Research Centre on Dynamics of High Latitude Marine Ecosystems (FONDAP-IDEAL), Casilla, 567 Valdivia Chile

**Keywords:** Carbon cycle, Marine chemistry

## Abstract

The role of appendicularian faecal pellet (FPa) size fractions on coccolithophore-derived particulate organic carbon (POC) and calcium carbonate (CaCO_3_) export to the deep sea was assessed from sediment traps within a period of ten years (1995–2004) off Coquimbo (CQ, 30°S) and five years (2005–2009) off Concepción (CC, 36°S) in the Humboldt Current System (HCS) off Chile. The composition and size distribution of 1,135 FPa samples from sediment traps deployed at 2,300 and 1,000 m depths showed non-linear, inverse relationships between the FPa size-fractions and their volume-specific POC and CaCO_3_ contents, which were up to ten times higher for small (<100 µm in diameter) than large (>100 µm) FPa. On average, 13 and 2% of the total POC and CaCO_3_ fluxes, respectively, were contributed mainly by small FPa (90%), with maxima during the autumn and summer. Thus, a non-linear, exponential model of volume-specific POC and CaCO_3_ contents of FPa substantially improved vertical flux rate estimates. In the HCS, annual carbon flux based on a non-linear FPa carbon load was double the estimate assuming a linear-volume to carbon load for FPa (345 and 172 kton C y^−1^). We recommend a widespread consideration of this non-linear model in global carbon estimates.

## Introduction

The Humboldt Current System (HCS) off Chile is one of the world’s most productive coastal upwelling systems^1^, originating from the West Wind Drift at circa 40–45°S^2^ and including the Coquimbo (30°S) and Concepción (36°S) upwelling centres. The coastal and oceanic regions between 30°S and 36°S are dominated by Sub-Antarctic Waters (SAAW) down to 150 m depth and approximately 700 nm westward. This part of the HCS is highly dynamic with a net northward flow of the SAAW, which bifurcates into two tongues (oceanic and coastal currents) and border the southward Chile-Perú counter current^3,4^. Below the SAAW down to 400 m depth lay the cold (<10 °C), nutrient rich, oxygen depleted (<1 mL L^−1^)^4^ Equatorial Subsurface Water^4^. Between 400 and 1,000 m depths predominate the Antarctic Intermediate Waters and >1,000 the Deep Pacific Water. The region between 30–36°S is characterized as a transition zone with high kinetic energy (>40 cm^2^ s^−2^) in a wide band parallel to the coast that can extend up to 800 km westward^5^.

The wind stress that favours upwelling along the coast off Chile predominates during spring-summer and contributes to fertilization (e.g. nitrate, phosphate) that promotes high primary production (>20 g C m^−2^ d^−1^)^1^ and phytoplankton biomass (>5 mg m^−3^) in the surface waters of the HCS^6^. The importance of the continental margins in the productivity and carbon cycles is widely recognized^7–9^; however, the little information on the function of the biological pump is restricted to specific upwelling centres within the vast area covered by the HCS^10–12^.

The biological carbon pump is an important component of the global carbon cycle in which the factors influencing zooplankton faecal material export, particularly PFa, remain poorly understood^13^. In the coastal areas of the HCS, the vertical flux of POC is driven mainly by diatoms and copepods^6^; however, in oceanic areas, the carbon flux is driven mainly by appendicularian/euphausiid faecal pellets and the carbonate flux by foraminifers and FPa loaded with coccolithophore shells. Thus, in oceanic areas of the HCS off Chile, appendicularians and coccolithophores are important functional groups in the carbonate pump^6,14,15^. Overall, the magnitude and efficiency of the total POC flux temporally and spatially depend on changes in zooplankton and phytoplankton community compositions, as well as on changes in the biological processes (i.e. zooplankton grazing rate, faecal pellet production rate, phytoplankton aggregation, microbial degradation) that affect the dynamics of particle flux from the euphotic zone in oceanic provinces^16^ and upwelling areas off central Chile^12^.

In the HCS, El Niño (EN) events show oceanographic changes such as intrusion of oceanic, low-nutrient, warm, oxygenated waters into the coastal areas that trigger positive sea-surface temperature anomalies. During EN events, the plankton assemblages shift to smaller species during the warm phase; however, the total phytoplankton biomass, primary production, and vertical flux of carbon do not change^3,17–19^. Conversely, during “La Niña” events, the shallowed nutricline leads to increased nutrient supply, phytoplankton biomass, and primary production^20^.

Several hundred sediment trap studies since the 1970s have reported the pivotal role of zooplankton faecal pellets (FP) in the carbon biogeochemical cycle^14,21–23^. This plethora of information demonstrates the importance of zooplankton FP in the export of particulate organic carbon (POC) from the photic zone to the deep sea^24,25^. The contributions of FP produced by different functional groups, including euphausiids, appendicularians, copepods, salps, and microzooplankton, to vertical flux of POC also have been reported^24–26^, where appendicularians stand out because of their high FP production rates (Supplementary Table S1) and carbon fluxes (Supplementary Table S2), but relatively low sinking rates (Supplementary Table S3). The high variability of those estimates seems to relate to many physical and biological interactions that affect export versus recycling of the carbon and nitrogen in FP. For example, appendicularians usually represent a minor fraction of total mesozooplankton biomass; however they can process large amounts of food in a short time and produce numerous FP that contribute to export production^27,28^, playing significant roles in the export of the POC^14^ and calcium carbonate (CaCO_3_) to the deep sea^6–29^ in many disparate areas of the world’s oceans.

**Figure 1:**
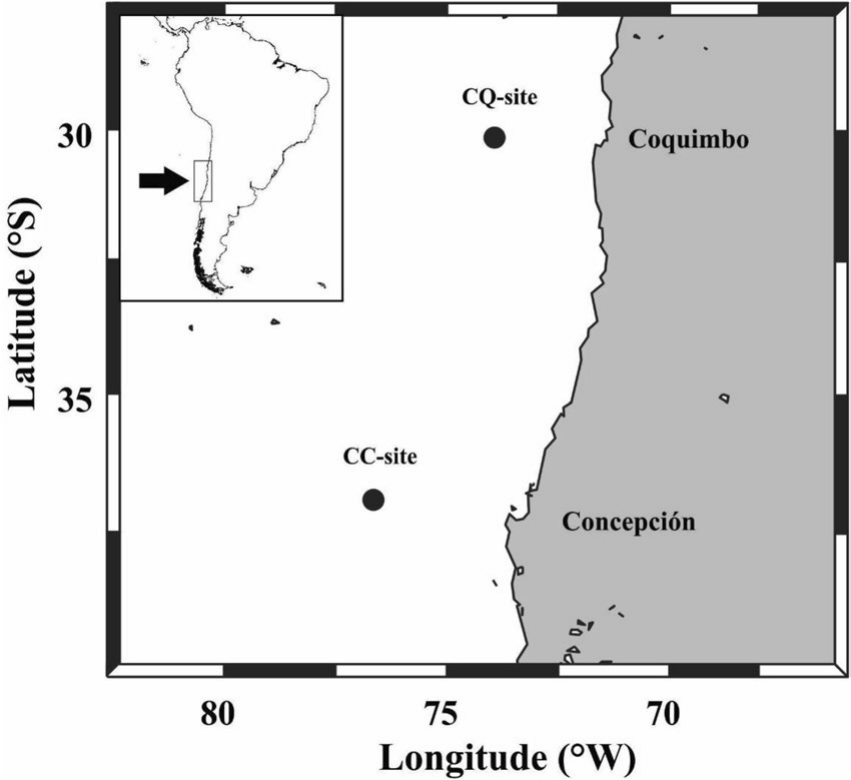
Locations of sediment traps deployed for determination of carbonate flux from appendicularian faecal pellets in the CQ (30°S) and CC (36°S) sites in the Humbolt Current System off Chile.

Coccolithophores also are an important component of the phytoplankton in the HCS off Chile and the world ocean^6,30,31^. Their transport to deep layers of the ocean has been principally by means of FPa, where a linear relationship between POC or CaCO_3_ contents with volume-derived FPa size has been assumed^32^. The reasoning is that the shells (coccoliths) increase the FPa sinking speed^33^, although there is little information on the appendicularian diets and the physical and chemical composition of FPa, which are usually assumed to be invariable and independent of the size of the animal and the extruded faecal pellet. We tested this assumption in order to determine the role of FPa of different sizes in the fluxes of POC and biogenic carbonate to the deep sea at two sites (CQ, 30°S; and CC, 36°S) (Table 1), in the HCS off Chile (Fig. 1).

**Table 1:** Station positions of the sediment traps off Coquimbo (CQ) and Concepción (CC) sites, trap depths, bottom depths at the trap locations, numbers of samples retrieved without problem, deployment periods and sampling intervals.

Station	Position		Trap depth (m)	Bottom depth (m)	Samples	Deployment period		Interval (d)
						Start	End	
CQ	30°S	73°11′	2,300	4,700	20	28 Jan 1995	22 May 1995	7
	30°S	73°11′	2,300	4,700	9	18 June 1995	14 Sept 1995	7
	30°S	73°11′	2,300	4,700	20	28, Jan 1996	28 June 1996	9
	30°S	73°11′	2,300	4,700	20	14 Feb1997	19 Oct 1997	14
	30°S	73°11′	2,300	4,700	18	15 Nov 1997	19 June 1998	13
	30°S	73°11′	2,300	4,700	20	11 Feb 1999	01 Aug 1999	10
	30°S	73°11′	2,300	4,700	6	14 Sept 2000	03 Dec 2000	17
	30°S	73°11′	2,300	4,700	15	15 Apr 2002	25 Nov 2002	17
	30°S	73°11′	2,300	4,700	12	05 Apr 2003	07 Oct 2003	18
	30°S	73°11′	2,300	4,700	5	22 May 2004	29 July 2004	18
CC	36°59′S	74°49′	1,000	4,400	16	12 Dec 2005	20 Sept 2006	19
	36°59′S	74°49′	2,300	4,400	20	18 Oct 2006	25 Sept 2007	19
	36°59′S	74°49′	2,300	4,400	20	27 March 2008	04 March 2009	19

Sample numbers less than 20 indicate periods when the sediment trap malfunctioned. The total samples obtained from sediment traps (CQ and CC) were 201. In the column “Samples”, the numbers <20 indicate malfunction of the sediment trap.

## Results

### FPa size-spectrum

**Figure 2:**
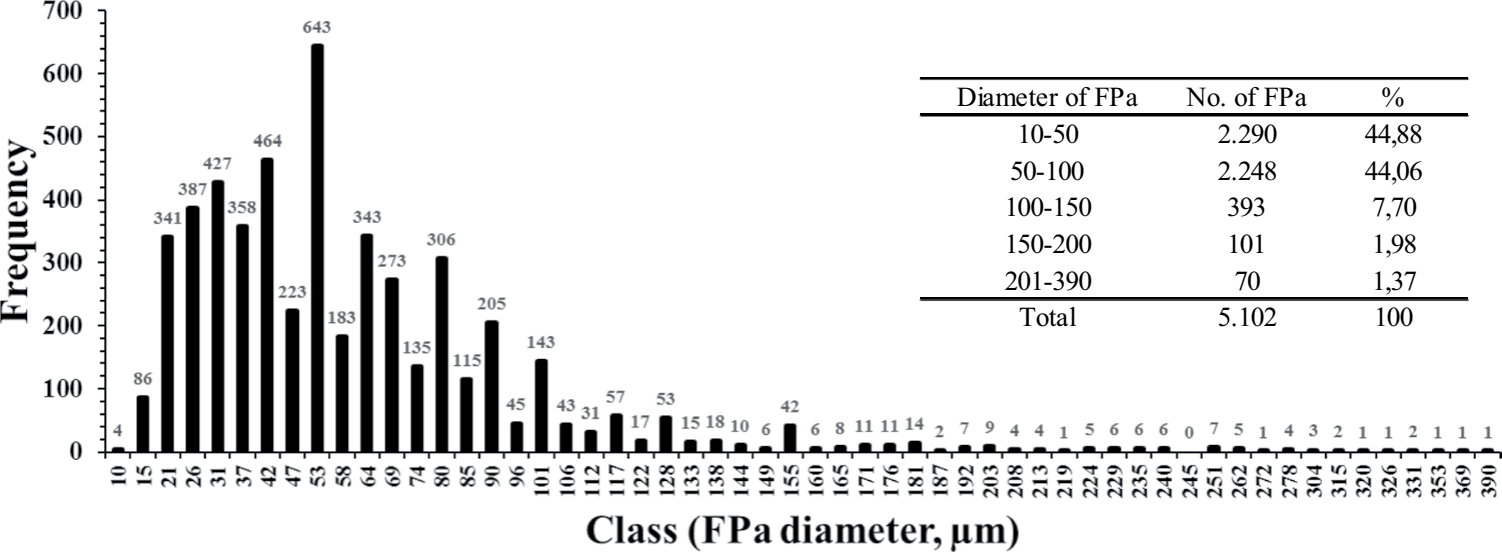
Frequency distribution of appendicularian faecal pellet (FPa) size fractions quantified from sediment trap samples deployed at the CC and CQ sites in the Humboldt Current System off Chile. The numbers of FPa per 5-µm size interval are shown above each bar. The numbers and percentages (%) of FPa in 50-µm size intervals are in the inset table. A total of 5,102 FPa were measured from 201 sediment trap samples (145 from the CQ and 56 from the CC sites).

The size-spectrum of FPa sizes (diameter and length) and volumes were analysed from 5,102 FPa isolated from 201 sediment trap samples from CQ and CC in the HCS, covering most of the study period. We found that 89% of the FPa were between 10 and 100 µm in diameter, highlighting the pivotal role of small FPa in fluxes of carbon and calcium carbonate in the oceanic region of the HCS off central Chile (Fig. 2). The remaining FPa (11%) were between 101 and 390 µm.

### Carbon and calcium carbonate contents in FPa

**Figure 3:**
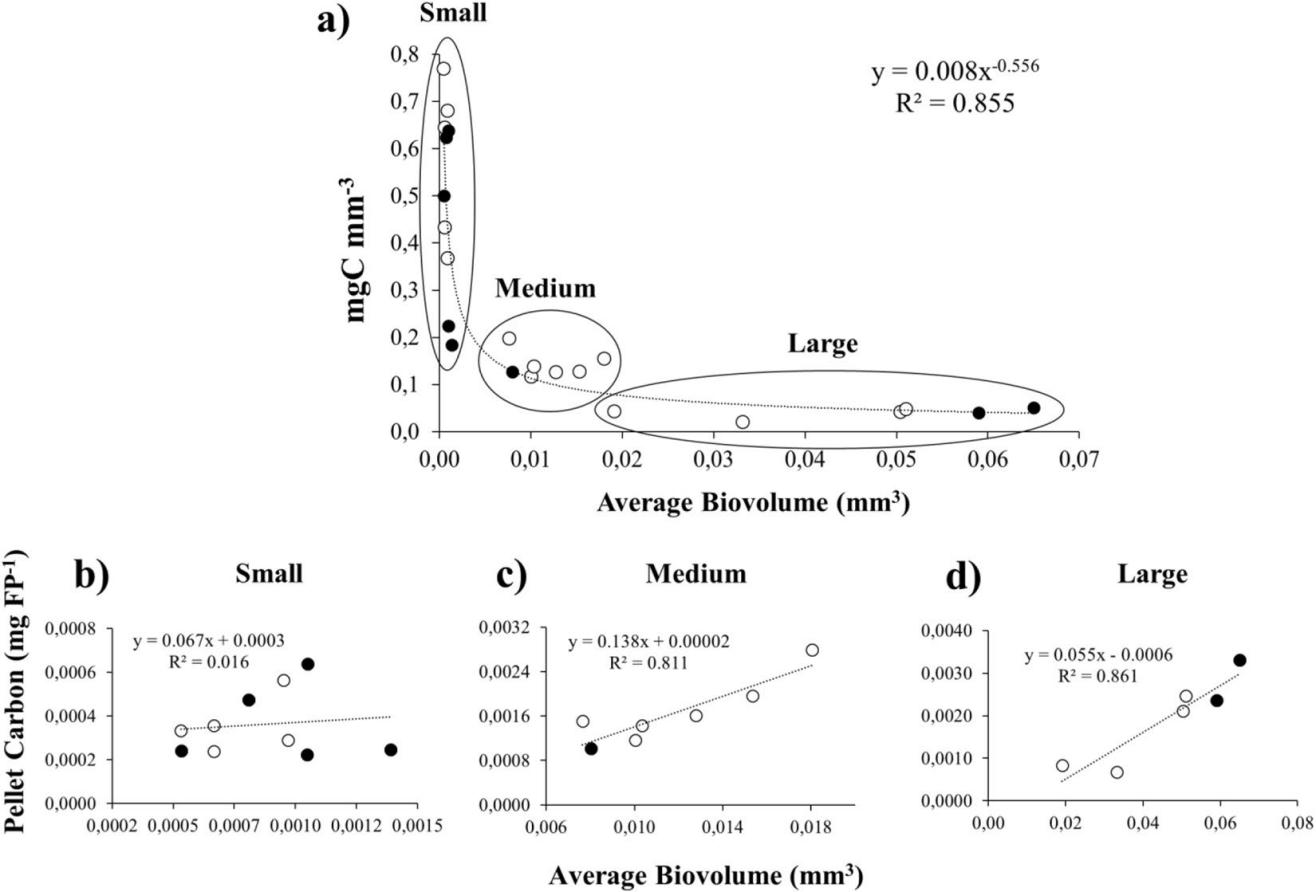
(**a**) Relationship between volume-specific carbon content (mg C mm^−3^) and average biovolume (mm^3^) for appendicularian faecal pellets (FPa) from the CQ (empty circles) and CC (filled circles) sites off Chile. (**b**–**d**) carbon contents of three different FPa size groups in relation to average biovolume: (**b**) small, (**c**) medium, and (**d**) large.

The inverse relationship between the FPa size fractions and their volume-specific POC content (mg C mm^−3^) was best represented by a power function (f(x) = 0.0087x^−0.556^; R^2^ = 0.85). This was obtained from measurements of 1,135 FPa isolated from sediment trap samples collected at stations CQ and CC during most of the study period. The POC in FPa was highly variable (0.02–0.77 mg C mm^−3^), with the highest volume-specific values (~0.2–0.77 mg C mm^−3^) in small FPa (Fig. 3a) and the lowest (0.02–0.2 mg C mm^−3^) in large FPa (Fig. 3). We also expressed the average concentration of carbon per FPa (mg C FPa^−1^) for each size group (small, medium, and large) as functions of their average biovolumes. Different slopes in each of the linear equations among the three size groups denote a highly non-linear and inverse relationships between FPa carbon content and their biovolumes in FPa (Fig. 3b–d).

### Coccolith abundance, CaCO_3_ content, and coccolithophore composition within FPa

**Figure 4:**
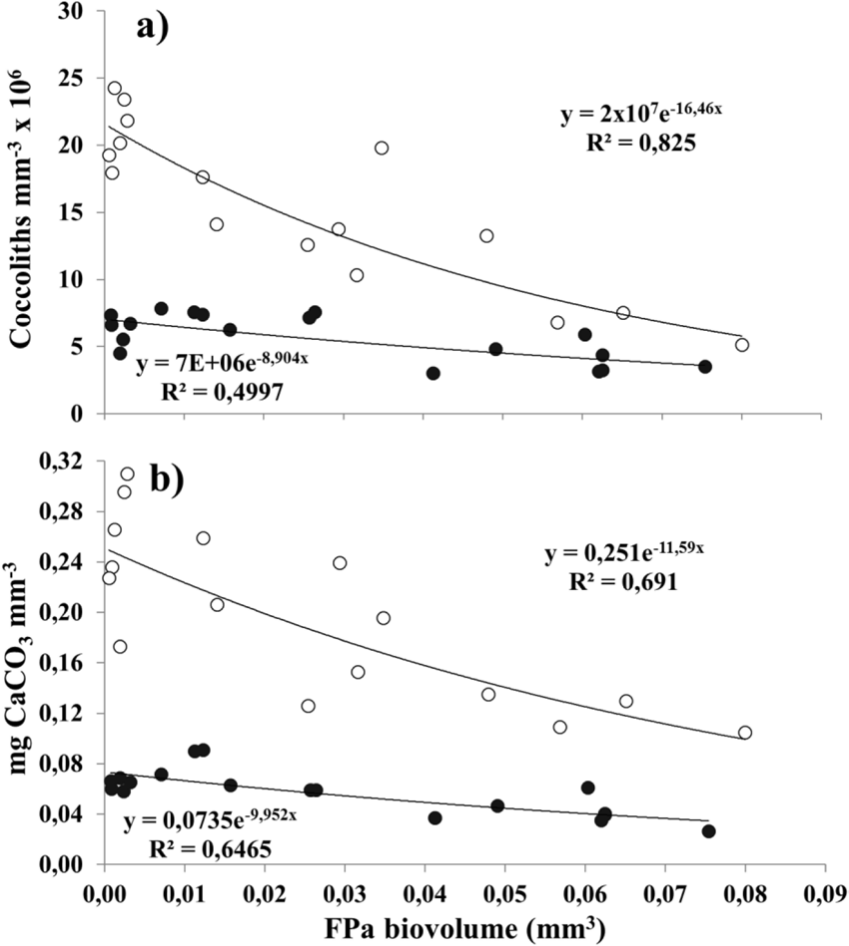
(**a**) Coccolith concentration (average number of coccoliths per mm^3^ × 10^6^) and (**b**) CaCO_3_ concentration in FPa (mg CaCO_3_ mm^−3^) as a function of appendicularian faecal pellets (FPa) biovolume (mm^3^) in samples from the CQ (empty circle) and CC (filled circle) sites off Chile.

The number of coccolithophore plates in the FPa matrix were quantified by species. The FPa ranged between 0.08–0.35 mm in diameter and 0.0006–0.080 mm^3^ in biovolume. The coccolith abundance and coccolithophore-derived calcium carbonate (CCa) concentration showed non-linear inverse exponential functions when related with its biovolume-specific (per mm^3^) value at both sites. At CQ, the mean coccolith and CCa contents were 5.1 to 24.3 × 10^6^ coccoliths mm^−3^ and 0.1 to 0.3 mg CaCO_3_ mm^−3^, respectively. The maximum biovolume-specific abundance of coccoliths and CCa were observed in the smallest FPa (2.1 × 10^7^ coccoliths mm^−3^; 0.24 mg CaCO_3_ mm^−3^) and the minimum in the largest (1.3 × 10^7^ coccolith mm^−3^; 0.18 mg CaCO_3_ mm^−3^) (Fig. 4a,b). In contrast, at the CC site, these parameters ranged from 7.8 × 10^6^ coccoliths mm^−3^ and 0.091 mg CaCO_3_ mm^−3^ to 2.9 × 10^6^ mm^−3^ and 0.027 mg CaCO_3_ mm^−3^, respectively. The smallest FPa showed higher coccolith abundance and CCa concentration (6.2 × 10^6^ coccoliths mm^−3^; 0.064 mg CaCO_3_ mm^−3^); in contrast, the largest FPa had the least (5.5 × 10^6^ coccoliths mm^−3^; 0.056 mg CaCO_3_ mm^−3^) (Fig. 4a,b).

On average, seventeen genera/species of coccolithophore were found in the FPa from both sites; however, there were important differences between the CQ and CC sites. The abundance of coccoliths and CCa concentrations were ~3 and 1.6 times higher in CQ than in CC site. The main coccolith type observed was *Emiliania huxleyi*, which represented >70% of the abundance of all recorded species and was ~3 times more abundant in CQ than the CC site.

The CCa concentrations in FPa were dominated by coccoliths belonging to the species *Calcidiscus leptoporus*, which contributed ~45% of total carbonate biomass, while *E. huxleyi* and *Helicosphaera carteri* together contributed ~40%. These three species represented >80% of the carbonate biomass in FPa in both sites (Table 2).

**Table 2:** Coccolithophore species found in the matrix of appendicularian faecal pellets (FPa) collected in sediment traps deployed at the CC and CQ sites off Chile and their contributions to total average carbonate and standard deviation (SD), The species-specific abundance of coccoliths per mm^3^ of FPa was counted in 35 bunches of measured (volume calculated) and sonicated FPa (16 from CQ and 18 from CC), The species-specific CaCO_3_ contents per coccolith (*) were taken from the literature^68^, The total coccolith abundance was depicted in Fig. 4.

Concepción (CC site)						Coquimbo (CQ site)						
Main	Coccoliths per FPa	SD Coccoliths per FPa	CaCO_3_ (pg) per coccolith (*)	CaCO_3_	SD CaCO_3_	CaCO_3_	Coccoliths per FPa	SD Coccoliths per FPa	CaCO_3_ (pg) per coccolith (*)	CaCO_3_	SD CaCO_3_	CaCO_3_
coccolithophore species	mm^3^	mm^3^		(mg µm^−3^)	(mg µm^−3^)	(%)	mm^3^	mm^3^		(mg µm^−3^)	(mg µm^−3^)	(%)
*Calcidiscus leptoporus*	388,977	142,845	79.5	1.8 × 10^−12^	2.2 × 10^−12^	46.955	1,144,288	412,790	79.5	2.7E-12	2.3 × 10^−12^	44.345
*Emiliania huxleyi*	4,610,351	1,535,543	2.36	7.8 × 10^−13^	9.6 × 10^−13^	20.728	11.942.614	5.212.936	2.36	1.1E-12	7.8 × 10^−13^	17.230
*Helicosphaera carteri*	80,238	33,545	138.9	7.1 × 10^−13^	1.2 × 10^−12^	18.876	254,033	147,097	138.9	1.2E-12	1.4 × 10^−12^	20.166
*Gephyrocapsa mullerae*	282,634	156,164	8.00	1.5 × 10^−13^	2.2 × 10^−13^	3.980	1,189,431	764,395	8.00	3.4E-13	4.6 × 10^−13^	5.512
*Gephyrocapsa oceanica*	56,616	44,208	20.2	8.0 × 10^−14^	1.7 × 10^−13^	2.110	389,992	350,497	20.2	1.6E-13	9.7 × 10^−14^	2.639
*Syracosphaera pulchra*	60,720	36,223	14.75	7.0 × 10^−14^	9.7 × 10^−14^	1.856	57,357	44,161	14.75	2.1E-14	1.8 × 10^−14^	0.337
*Helicosphaera spp*,	5,084	4,751	138.9	5.5 × 10^−14^	1.0 × 10^−13^	1.445	44,502	38,867	138.9	1.6E-13	1.3 × 10^−13^	2.568
*Radbosphaera clavigera*	7,335	4,492	67.5	4.7 × 10^−14^	8.0 × 10^−14^	1.252	23,863	28,640	67.5	3.2E-14	3.1 × 10^−14^	0.527
*Scyphosphaera apsteinii*	996	2,071	540	3.7 × 10^−14^	1.0 × 10^−13^	0.979	6,577	8,000	540	1.4E-13	1.9 × 10^−13^	2.280
*Umbilicosphaera sibogae*	25,610	13,103	17.3	3.5 × 10^−14^	5.7 × 10^−14^	0.928	363,557	223,348	17.3	2.2E-13	2.6 × 10^−13^	3.570
*Coccolithus pelagicus*	1,332	1,207	130	1.9 × 10^−14^	3.3 × 10^−14^	0.491	463	679	130	3.6E-15	6.3 × 10^−15^	0.058
*Pontosphaera syracusana*	1,617	1,693	65.9	1.0 × 10^−14^	1.5 × 10^−14^	0.267	4,057	6,379	65.9	7.9E-15	7.2 × 10^−15^	0.128
*Florisphaera profunda*	9,862	42,986	0.17	6.2 × 10^−16^	2.7 × 10^−15^	0.016	764,319	362,900	0.17	3.9E-14	3.5 × 10^−14^	0.635
*Gephyrocapsa mullerae (coccosphere)*	770	1,865	160	4.2 × 10^−15^	1.2 × 10^−14^	0.111	0	0	160	0	0	0.000
*Calciosolenia murrayi /brasiliensis*	6,718	8,216	0.1	4.4 × 10^−17^	5.6 × 10^−17^	0.001	31,701	34,090	0.1	1.1E-16	1.4 × 10^−16^	0.002
*Gaaderia corolla*	1,665	2,255	0.1	1.3 × 10^−17^	2.1 × 10^−17^	0.000	0	0	0.1	0	0	0.000
*Syracosphaera anthos*	8,311	5,583	0.1	6.2 × 10^−17^	8.4 × 10^−17^	0.002	22,640	20,210	0.1	9.4E-17	1.3 × 10^−16^	0.002
*Syracosphaera spp*,	24,690	26,425	0.1	1.1 × 10^−16^	1.2 × 10^−16^	0.003	33,490	48,167	0.1	1.3E-16	2.9 × 10^−16^	0.002

### POC, PIC/POC ratios and CaCO_3_ fluxes mediated by FPa in CQ and CC

**Figure 5:**
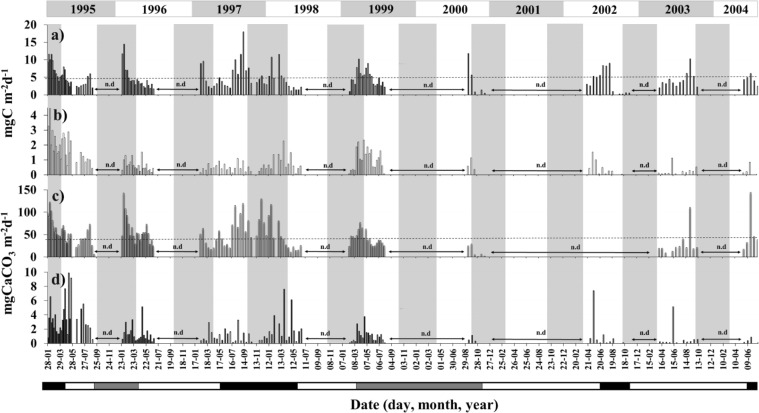
(**a**) Total particulate organic carbon (POC) flux (mg C m^−2^ d^−1^) within the period 1995–2004 off Coquimbo (30°S) at 2,300 m depth. (**b**) Appendicularian faecal pellet (FPa)-mediated POC flux derived from a FPa non-linear, volume-specific POC model. (**c**) Total carbonate flux and (**d**) FPa-mediated carbonate flux (mg CaCO_3_ m^−2^ d^−1^). The dashed line in (**a**) represents the mean total POC flux (4.7 mg C m^−2^ d^−1^) and the dashed line in (**c**) represents the mean total carbonate flux (48.1 mg CaCO_3_ m^−2^ d^−1^). The top bar represents the period between 1995 and 2004 while the vertical shading in gray represents the spring-summer periods. The bar at the bottom shows “El Niño” (black), “La Niña” (gray), and “normal” (white) periods (www.cpc.noaa.gov/products/analysis_monitoring/ensostuff/ensoyears.shtml). n.d. along the horizontal axes show periods of no data due to equipment failure.

**Figure 6:**
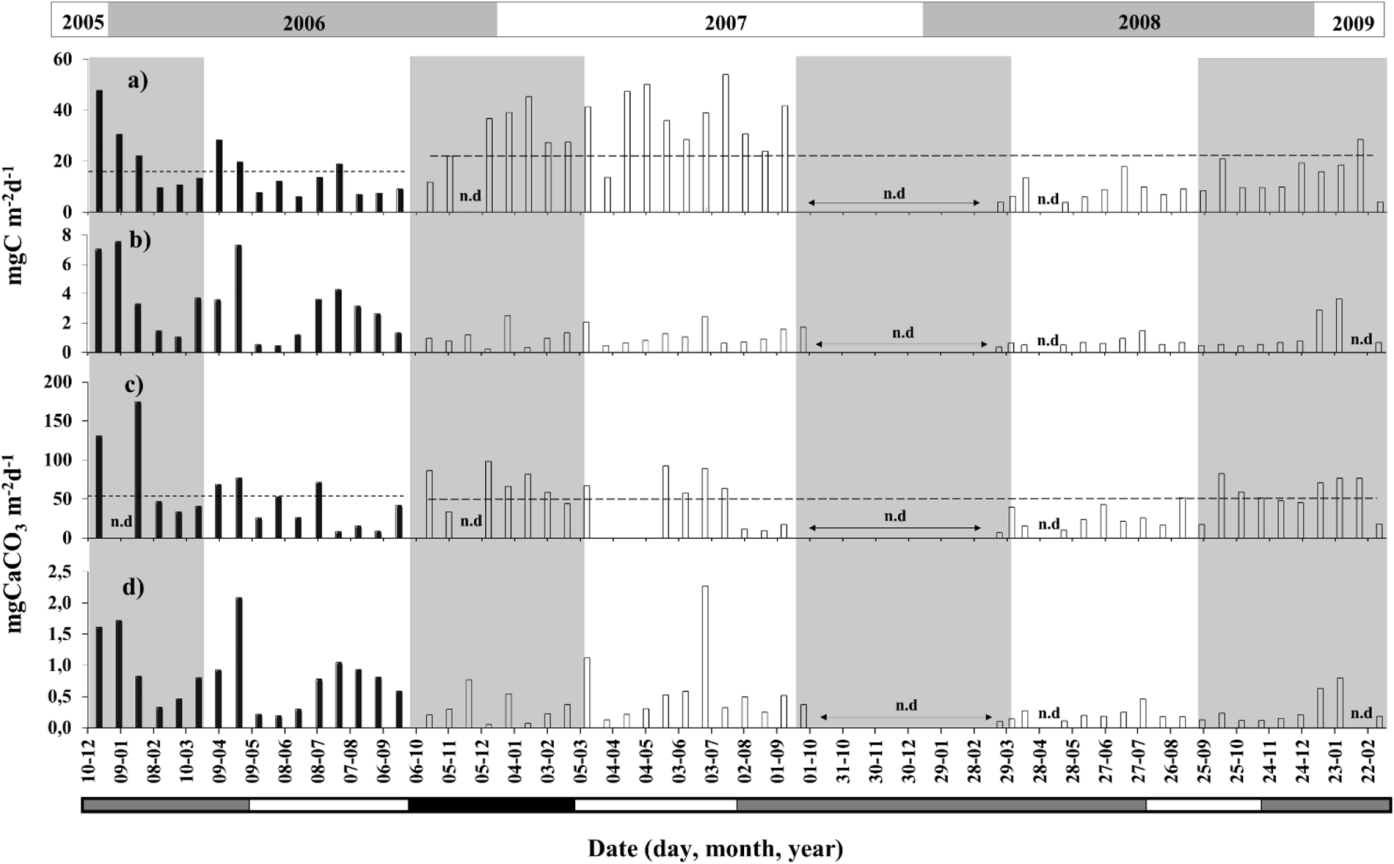
(**a**) Total particulate organic carbon (POC) flux estimated in sediment traps deployed in CC at 1,000 m depth (20/Dec/2005 to 20/Sept/2006, black bars) and 2,300 m depth (22/Sept/2006 to 04/Mar/2009, empty bars); (**b**) appendicularian faecal pellet (FPa)-mediated POC flux (mg C m^−2^ d^−1^); (**c**)Total carbonate flux and (**d**) FPa-mediated carbonate flux (mg CaCO_3_ m^−2^ d^−1^). The top bar represents the period between 2005 and 2009 while the vertical shading in gray represents the spring-summer periods. The bar at the bottom shows “El Niño” (black), “La Niña” (gray), and “normal” (white) periods. The dashed line upper of the black bar in (**a**) represents the mean total POC flux (15.6 mg C m^−2^ d^−1^) to 1,000 m of depth, while the dashed line upper of the empty bar show of the average of the 2,300 m of the depth. The dashed line in (**c**) represents the mean total carbonate flux (51.6 mg CaCO_3_ m^−2^ d^−1^) to the 1000 m of depth, while that the dashed line upper the empty bar showed the mean 44.5 mg CaCO_3_ m^−2^ d^−1^ of the 2,300 m of depth. n.d. along the horizontal axes show periods of no data due to equipment failure.

Fluxes of POC and CaCO_3_ in FPa at 2,300 and 1,000–2,300 m depths in the oceanic region off CQ and CC varied greatly during time series stations measured (with gaps due to malfunctions of the traps) in time periods of ten and five years (1995–2004 and 2005–2009), respectively (Figs. 5 and 6). Overall in the CQ site, the FPa-mediated fluxes averaged 18.1% of the total POC flux (average 4.7 mg m^−2^ d^−1^) (Fig. 5a,b) and 3.4% of the total FPa-mediated CaCO_3_ flux (48.1 mg m^−2^ d^−1^) (Fig. 5c,d).

In the CC site, FPa-mediated fluxes averaged 19.8% (3.1 × 100)/15.6) and 4% (0.86 × 100)/21.36) of the total POC flux to the 1,000 m (average 15.6 mg C m^−2^ d^−1^) and 2,300 m (average 21.4 mg C m^−2^ d^−1^), respectively. While that the general average was 7.7% of the total POC flux (19.7 mg C m^−2^ d^−1^) (Fig. 6a,b and Table 3).

**Table 3:** POC, CaCO_3_, C-CaCO_3_ (mgC m^−2^d^−1^) and PIC/POC ratios estimated from deep sediment traps (range 1,000–3,700 m depth) deployment in different areas of the world oceans.

Study area	Depth sediment trap (m)	POC flux mg m^−2^d^−1^	CaCO_3_ flux mg m^−2^d^−1^	C-CaCO_3_ mg m^−2^d^−1^	PIC/POC ratios	Reference
Panama Bight (5°22′N, 85°35′W)	2,590	11.20	184.20	22.10	1.97	Honjo (1982)^45^
Station P (501 N; 1451 W)	3,800	10.50	67.90	8.15	0.78	Honjo (1984)^46^
Arabian sea (14.51 N; 651 W)	2,800	1.20	11.82	1.42	1.18	Nair *et al*.^47^
Equatorial Pacific (121 S; 135°W)	3,600	0.70	16.00	1.92	2.74	Honjo *et al*.^48^
Bay of Bengal (131 N; 841E)	2,300	6.70	42.13	5.06	0.75	Ittekkot *et al*.^49^
Humboldt current (30°S 73°11′W)	3,700	7.50	94.70	11.36	1.61	Hebbeln *et al*.^3^
Humboldt current (30°S 73°11′W)	2,300	7.50	82.39	9.89	1.69	Hebbeln *et al*.^3^
Subantarctic zone (53°02′S; 174°44′W)	1981	2.70	18.60	2.23	0.83	Honjo *et al*.^50^
Subantarctic zone (60°17′S; 170°03′W)	1,003	6.30	35.10	4.21	0.67	Honjo *et al*.^50^
West Sargasso Sea (31°50′N; 64°10′W)	3,200	2.40	22.40	2.69	1.12	Conte *et al*.^51^
West Sargasso Sea (31°50′N; 64°10′W)	1,500	1.70	21.00	2.52	1.48	Conte *et al*.^51^
East China Sea slope (25°11′N;122°58′E)	1,340–1,588	128.60	214.20	25.70	0.20	Hung *et al*.^52^
Humboldt current (30°S 73°11′W)	2,300	7.00	61.00	7.32	1.05	González *et al*.^14^
Arabian Sea (16°18′N 60°30′E)	1,200	14.80	106.80	12.82	0.87	Ramaswamy *et al*.^53^
Bay of Bengal (13°09′N 84°20′E)	2,282	8.50	39.50	4.74	0.56	Ramaswamy *et al*.^53^
Arabian Sea (03°34′N 77°46′E)	2,394	4.40	49.80	5.98	1.36	Ramaswamy *et al*.^53^
Bay of Bengal (05°01′N 87°09′E)	3,010	5.70	37.80	4.54	0.80	Ramaswamy *et al*.^53^
Humboldt current (30°S 73°11′W)	2,300	4.74 (0.86*)	48.12 (1.64*)	5.77 (0.19*)	1.22 (0.22*)	This Study
Humboldt current (37°05′S 74°50′W)	1,000	15.60 (3.10*)	51.62 (0.81*)	6.19 (0.097*)	0.39 (0.031*)	This Study
Humboldt current (37°05′S 74°50′W)	2,300	21.36 (0.86*)	44.54 (0.32*)	5.34 (0.038*)	0.25 (0.044*)	This Study
Humboldt current (37°05′S 74°50′W)	1,000/2,300	19,65 (1.51*)	46,66 (0.47*)	5.60 (0.056*)	0.28 (0.037*)	This Study

C-CaCO_3_ correspond to inorganic carbon obtained from CaCO_3_ bulk. (*) contribution of the FPa to the carbon and carbonate flux in the CQ and CC site in the HCS.

The total FPa-mediated CaCO_3_ flux represented in average the 1.6% (0.81 × 100)/51.6) and 0.7% (0.32 × 100)/44.54) to the 1,000 m (average 51.6 mg CaCO_3_ m^−2^ d^−1^) and 2,300 m (average 44.5 mg CaCO_3_ m^−2^ d^−1^), respectively. However, considering both depth (1,000 and 2,300 m) the average was ~1% of the total FPa-mediated CaCO_3_ flux (46,7 mg CaCO_3_ m^−2^ d^−1^) (Fig. 6c,d).

The total PIC/POC ratios for the CQ site (1.2) was higher than for CC site (0.28). On the other hand, the PIC/POC ratio was slightly higher in the FPa from CC-site (0.037, from both depths), than for CQ-site (0.022), respectively (Table 3).

In the CQ site, maximum fluxes of FPa-mediated POC ranged between 2.3 and 4.4 mg C m^−2^ d^−1^ and occurred during summer-autumn 1995 (Fig. 5b), while in the CC site, the maximum values (3.0–7.4 mg C m^−2^ d^−1^) occurred during the summer-autumn 2005–2006 and winter of 2006 (Fig. 6b). Similarly, fluxes of FPa-derived CaCO_3_ for the CQ site were highest in summer-autumn of 1995, 1998, and 2002 (6.0–9.8 mg CaCO_3_ m^−2^ d^−1^) (Fig. 5d), while high fluxes for the CC site (1.5–1.6 mg CaCO_3_ m^−2^ d^−1^) occurred during the summer-autumn of 2005–2006 (Fig. 6d).

The average annual FPa-mediated POC fluxes in the CQ site were highest during 1995 (1.78 mg C m^−2^ d^−1^) and 1999 (1.04 mg C m^−2^ d^−1^) and lowest (0.22 mg C m^−2^ d^−1^) during 2003 (Fig. 5d). For the CC site, the highest fluxes were recorded during 2005 (6.9 mg C m^−2^ d^−1^) and 2006 (2.4 mg C m^−2^ d^−1^) (Fig. 6b). Likewise, the average annual FPa-mediated CaCO_3_ fluxes were highest during 1995 for the CQ site (3.5 mg CaCO_3_ m^−2^ d^−1^) and 2005 for the CC site (1.5 mg CaCO_3_ m^−2^ d^−1^); and lowest during 2004 (0.32 mg m^−2^ d^−1^) and 2008 (0.1 mg m^−2^ d^−1^).

For the CQ site, the FPa-mediated POC flux during 1995 (1.78 mg C m^−2^ d^−1^) was statistically different (Mann-Whitney test, n = 16, p < 0.01) from all other years combined; similarly, 1996–1998 (0.6–0.8 mg C m^−2^ d^−1^) was statistically different from 2002–2003 (0.3–0.2 mg C m^−2^ d^−1^) (Mann-Whitney test, n = 13, p < 0.05). High seasonal variability in FPa-mediated fluxes of POC and CaCO_3_ was observed, with maxima in summer/autumn (1.2 and 2.1 mg m^−2^ d^−1^) and minimum values in spring (0.4 and 0.6 mg m^−2^ d^−1^, respectively). Fluxes differed statistically (Mann-Whitney test, n = 31, p < 0.05) between summer and winter-spring and between autumn and winter-spring.

For the CC site, fluxes of FPa differed significantly (Mann-Whitney test, N = 18, p < 0.05) among all years, except when we compared the pairs 2006–2009 and 2007–2009 that were similar (Fig. 6b,d). No significant seasonal differences in FPa-mediated POC or CaCO_3_ were observed (Mann-Whitney test, N = 35, p > 0.05) during El Niño, La Niña, or “normal” periods (see Figs 5 and 6) in either of the two study areas (Mann-Whitney test, N = 11, p > 0.05).

## Discussion

The Biological Carbon Pump is an important component of the global carbon cycle in which the factors influencing zooplankton faecal material export efficiency and its controlling factors, particularly for PFa export, remain poorly understood. Overall, the magnitude and efficiency of the total POC flux in time and space depend on changes in the zooplankton and phytoplankton community compositions, as well as on changes in biological processes (i.e. zooplankton grazing rate, FP production rate, phytoplankton aggregation, microbial degradation) that affect the dynamics of particle flux from the euphotic zone in oceanic provinces^16^ and upwelling areas off central Chile^11^. The bulk of the faecal material usually is removed quickly from the upper water column due to microbial degradation and zooplankton activity^34,35^, although zooplankton faecal material still is a prominent fraction of total POC export in many areas of the world oceans^13,14^.

The composition of the faecal material collected in sediment traps deployed at the CQ and CC sites was represented mainly by three functional groups: appendicularians, euphausiids, and undetermined (where high degradation, amorphous or broken pieces precluded classification).

Published estimates of the POC content of FPa and its contribution to carbon export from the HCS have assumed an average carbon:volume ratio of 0.048 mg C mm^−3^ ^14,32^, but we demonstrated in this study that FPa size and both carbon and carbonate contents were related non-linearly (Figs 3a, 4b). For example, the mean carbon content per mm^−3^ in small FPa was five times higher than in large FPa (0.26 and 0.041 C mm^−3^, respectively). This result is important for quantitative estimates of the biological pump intensity, assuming that the PFa contents would be released in the same proportions as found in the sediment traps in this study, where more than 90% of the FPa were small. Our exponential conversion increased the contribution of the FPa-mediated carbon flux by ~50% (1.04 mg C m^−2^ d^−1^) over that obtained using a linear conversion factor between carbon content and FPa-size^14^.

The calcareous components found in the matrix of FPa from the CQ and CC sites were mainly coccolith plates (mean 10.3 × 10^6^ plates mm^−3^, Fig. 4A), from which the most abundant coccoliths were *Calcidiscus leptoporus*, *Helicosphaera carteri* and *Emiliania huxleyi*, which together averaged 86% of the CCa or 2.4 × 10^−13^ mg CaCO_3_ µm^−3^ in PFa isolated from sediment trap samples deployed at the CQ and CC sites (Table 2).

While existence of coccoliths and coccospheres embedded in the matrix of FPa have been reported^28^, but no prior quantitative information on their contribution to carbonate flux in the ocean has been previously made. The coccoliths and coccospheres within the matrices of FPa are important in the export of carbonate and increase the sinking velocity of FPa to the deep ocean. Previous work has demonstrated that 1-mm particles that are 90% carbonate by mass (and 10% POM) sink ~fivefold faster than particles that are 60% carbonate by mass (and 40% POM) and ~1.75 times faster than particles that are 90% silicate by mass (and 10% POM)^33^. An individual coccolith has a sinking velocity of 0.1 m d^−1^ or slightly higher, depending on species types and calcification degree. For example, *E. huxleyi*, *G. oceanica*, and *C. leptoporus* coccolith settling velocities (0.3, 0.7, and 4.3 m d^−1^, respectively) were higher than diatom sinking rates, which ranged from 0.07 m d^−1^ (*Thalassiosira weissflogii*) to 0.2 m d^−1^ (*Thalassiosira oceanica*)^36^. This information clearly suggests that FPa loaded with coccoliths and coccospheres can reach a higher sedimentation velocity than one loaded with diatom frustules, demonstrating the effectiveness of coccoliths and coccospheres in the transport of carbon and carbonate to the deep ocean.

The absence of a linear relationship between FPa-size and POC and CaCO_3_ content may be attributable to differences in the size spectrum of the food ingested, with small FPa being more uniform and compact than large PFa. To test this hypothesis, we isolated large and small FPa from a sediment trap deployed at 2,300-m depth during different seasons between 1995 and 2004 and analysed the particle content qualitatively. Large FPa contained many relatively large pieces of diatom frustules (20–63 µm diameter), mostly from the genus *Pseudo-nitzschia*, but also from the abundant *Thalassiosira* spp. and *Planktoniella sol*. In addition, the remains of radiolarians (groups Spummellaria, Nasselaria, and Phaeodaria), tintinnid loricae (genera *Codonellopsis*, *Dictiocysta*, *Protorhabdonella*, *Undella*, and *Dadayiela*), and silicoflagellates, such as *Dictiocha fibula*, were frequently observed. In contrast, microscopic analysis of small FPa demonstrated a preponderance of coccoliths, with occasional remains of diatoms and tintinnid loricae embedded in the FPa matrix. Large oikopleurid species have food-concentrating filters with a coarser mesh than small ones^37^, suggesting a direct relationship between pore dimensions of appendicularian filters and body size. Our study suggested that large appendicularians utilized a broad food-size spectrum (diatoms, coccolithophores, tintinnids, radiolarians, etc.), while the diet of small appendicularians was more restricted, which partially explained the small mineral skeletons in small FPa.

The FPa biovolume to POC (or CaCO_3_) content models that should be used to estimate the role of FPa in biomineral vertical fluxes follow size-dependent non-linear relationships, indicating that the FPa size fraction needs to be considered to improve the accuracy of these estimates. We strongly recommend a more taxa-specific analysis by microscopy, which is more time consuming but essential when dealing with size-dependent FPa-mediated POC and carbonate export processes. This view is supported by findings of changing composition of sinking particles across a region with unchanging carbon flux, suggesting that variability in the mechanisms of carbon flux^38^ or the role of phytoplankton biominerals as ballast^39^ may not be reflected by bulk measurements. In addition, coccolithophore calcium carbonate is usually considered to have a low carbon flux and high export efficiency^24^. Thus, CCa is generally not included in calculations of FPa carbon export, resulting in up to 4% underestimation of total carbon flux to deeper areas of the HCS.

High seasonal and inter-annual variability in POC flux was observed in the time series station off the CQ site (Fig. 5). The estimated average FPa-driven POC flux at the CQ site (0.86 mg C m^−2^ d^−1^) was slightly higher than that reported for the same area during 1993–1995 (~0.7 mg C m^−2^ d^−1^)^14^, while that recorded for the CC site was substantially higher (1.5 mg C m^−2^ d^−1^) than at the CQ site (Figs 5b, 6b).

Overall, the use of the FPa-size-based carbon content model of this study increased the estimated contribution of FPa to total POC export by a factor of 34% (CC site) and 46% (CQ site) over those obtained from a linear FPa-volume to carbon content model.

The highest average FPa flux occurred during the productive summer period, coinciding with intensification of the winds favouring upwelling and Ekman transport^14^, which may enhance local productivity and carbon export. We did not find significant differences (p > 0.05) in the PFa-mediated fluxes of POC or CaCO_3_ among El Niño, La Niña, and “normal” periods (showed in Figs 5 and 6), suggesting that the diet of the appendicularians was not severely affected by these events.

Reports during the El Niño event in the HCS show that the seasonal upwelling ceases, the water column became warmer, and the thermocline and nutricline deepens significantly during the passage of coastal-trapped waves^40,41^. These events decrease phytoplankton productivity and alter the trophic chain, changing the biological composition and physical dynamics of the coastal and marine ecosystems^18,41^. Both phytoplankton and zooplankton shift toward small-sized species that have been claimed to prevail during El Niño^17,18,40^. Thus, it is highly probable that the composition and size spectrum of small food particles used by appendicularians, down to colloids (<0.2 µm)^42^, is little affected by El Niño events^37,43^, a situation that might be extrapolated to future global warming scenarios on the FPa-mediated carbon and CaCO_3_ export fluxes.

**Figure 7:**
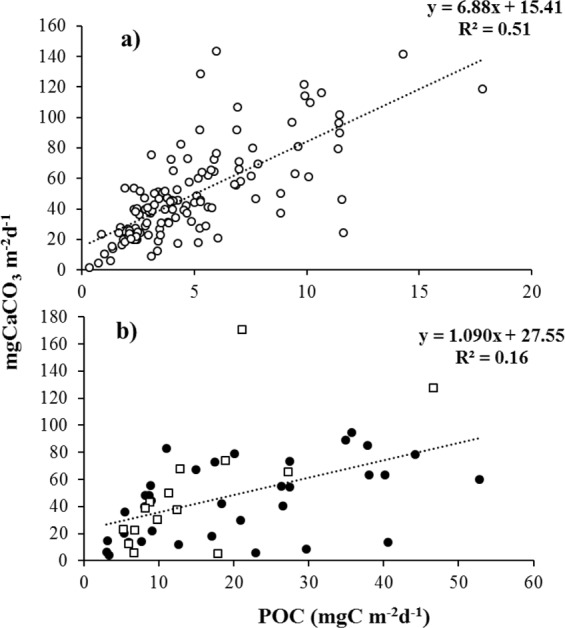
Linear correlations between total CaCO_3_ and total POC fluxes obtained from sediment trap samples deployed at 2,300 m depth at the CQ site (**a**) and 1,000 (empty square) and 2,300 (black circles) m depths at the CC site (**b**) off Chile.

For the CQ site, fluxes of calcium carbonate and POC into sediment traps below 2,300 m were correlated (y = 6.889x + 15.417, n = 127; R^2^ = 0.51) (Fig. 7a), which is consistent with published data for various geographic regions^3,14,44–53^, like the PIC/POC ratios (Table 3). Some of these oceanic and coastal areas (such as the CQ site) exhibit relatively low productivity^1^ that favours the export of CaCO_3_ (mean 48.1 mg m^−2^ d^−1^) over POC (mean 4.7 mg m^−2^ d^−1^) (Fig. 5a,c) and possibly carbonate-driven POC export as the main ballast mechanism, which would explain the high PIC/POC ratio (>1) observed in this site. Thus, biominerals increase substantially sinking velocity because of their high densities and may serve to protect organic matter from degradation during transit to the ocean floor^53–55^. Conversely, POC and CaCO_3_ at CC, were not significantly correlated (y = 1.090x + 27.56, n = 30; R^2^ = 0.16) (Fig. 7b), and the PIC/POC ratio was lower (<1) than the CQ site, which may be attributable to a very productive upwelling centre that supports massive diatom blooms^1^ and favours the export of both CaCO_3_ (mean 46.7 mg m^−2^ d^−1^) and POC (mean 19.7 mg m^−2^ d^−1^) (Fig. 6a,c) with perhaps silicate-driven POC export as the main ballast mechanism.

In the HCS, an average appendicularian abundance is estimated at 21.6 ind. m^−3^ ^27,56^ or 1,078 ind. m^−2^ integrated down to 50 m depth (see www.st.nmfs.noaa.gov/copepod/atlas). On average, one appendicularian can produce between 7.3 and 10.1 FPa h^−1^ ^27,57^ or an average of 198.6 FPa d^−1^ or 72,500 FPa y^−1^ (Supplementary Table S4). We estimated a total area of the HCS off Chile of 910,000 km^2^, assuming a total latitudinal length of 2,600 km between 18.5° and 41.5°S and a width of 350 km^58^. Because most appendicularians occur within the upper 50 m of the water column^59,60^, an estimate of 1.08 × 10^9^ appendicularians within this upper layer would produce 7.12 × 10^19^ FPa y^−1^ along the whole HCS. Moreover, considering the size-spectrum of FPa found at both study sites (Fig. 2), we assumed that ~90% of the FPa produced are small FPa (<100 µm of diameter) and 10% large. As the small FPa had an average biovolume and carbon content of 0.26 mg C mm^3^ and 0.000364 mg C, respectively, while equivalent values for a large FPa were 0.041 mg C mm^3^ and 0.0015 mg C (Fig. 3a and Supplementary Table 4). Finally, we estimated that 3.4 × 10^16^ mg C y^−1^ or 0.034 Gt C would be released annually as FPa in the upper 50 m water column of the HCS (Supplementary Table S4).

Given that the FPa vertical flux averaged 1.04 mg C m^−2^ d^−1^ (Figs 5b, 6b), we can estimate that 345.5 × 10^6^ kg carbon (or 0.000345 Gt carbon) are exported annually down to 2,300 m depth in the HCS, which represents 1% of the FPa carbon standing stock within the upper 50 m of the water column, suggesting extensive recycling of FPa between the photic zone and deeper layers of the HCS (Supplementary Table 4). We lack data on POC fluxes and degradation rates between 2,300 m of depth and the sea floor (circa 4,500 m depth, in CQ and CC), but information on POC recycling in many disparate regions of the world oceans indicate that an additional POC loss of ~30% might occur^61^. This suggests that ~0.7% of the standing stock of the FPa-mediated carbon export from the upper 50 m stratum could reach the ocean floor along the HCS off Chile.

This is in line with reports suggesting that <3% of the generated upper production reaches bathypelagic depths (>1,000 m) in the deep sea^62^, mainly due to the highly complex and variable food web in the water column^63^ that removes most of the sinking particles, such as the FPa.

Our non-linear FPa-size derived carbon to volume ratio estimated an average POC flux in the HCS off Chile to be 345 kton C y^−1^, twice as much the estimated with the linear model (172 kton C y^−1^). This study only includes the HCS off Chile, a very productive ecosystem that might differs from the rest of the global oceans (in food supply quality and quantity). Nevertheless, a widespread characteristic of the model is its non-linear, FPa-size derived nature, that we recommends to be considered for future estimated where FPa are key vehicles of PIC and/or POC carbon export.

## Methods

### Analysis of total POC and CaCO_3_

Samples were obtained from sediment traps (SMT 230, Salzgitter Electronic, Kiel, equipped with 20 sample bottles) deployed at 2,300 m depth ~150 km off the CQ site (30°S, off Coquimbo city). At the CC site (36°, off Concepción city), the trap was initially deployed down to 1,000 m depth (2005–2006), but later moved down to 2,300 m depth (2006–2009). The water depths in the study sites were 4,700 m for CQ and 4,300 m for CC (Table 1). Sample collection cups were changed between 7 to 18 days in the CQ and 19 days for the CC site (Table 1). Before deployment, each collection cup was filled with a hypersaline NaCl–seawater solution (38–40 g kg^−1^, prepared with water collected at 2000 m and 1% (v/v) saturated HgCl_2_) to retard bacterial activity in the trap material^64^. After recovery, the samples were poisoned again with HgCl_2_ (0.5 mL/100 mL of seawater) and stored at 4 °C. In the laboratory, a fraction of the samples was split into aliquots by a rotary liquid splitter. The splitting procedure, sample preparation, and analysis were previously described^65^. Subsequently, particulate organic carbon (POC) analyses were done after removal of CaCO_3_ with HCL 2 N, while for total particulate carbon (TPC), part of the sample (e.g. 1/20 to 1/4,096) was filtered (pre-combusted GF/F Whatman) and dried at 50 °C. Later, these samples were analysed in a Carlo Erba C/N-analyser using acetanilide as a standard^6^. Finally, the TPC and POC data were converted by a stoichiometric balance for the final determination of the total particulate carbonate ((TPC-POC) x 8.3333)^3^.

### Analysis of POC in FPa

Intact FPa (1,114 units, 528 from CQ and 586 from CC) of various sizes between 50 and 340 µm in diameter were isolated from sediment trap samples that covered most of the study period, and measured (major and minor axes of the FPa ellipsoid shape) by using a stereo microscope (Leica model MZ6). After a gentle wash with de-carbonated mineral water, using petri dish, to maintain the integrity of the FPa and photographed (Nikon Coolpix model 4500) using the stereo-microscope, then they were placed on 23 Whatman GF/F glass fibre filters in batches depending on their size (from 14 large to 142 small FPa). The images were processed in ImageJ software to estimate the volume of each faecal pellet and the total volume of each batch. Finally, subsamples of FPa on GF/F filters were acidified with 0.2 mL (~4 drops with a Pasteur pipette) of 2 N HCl to remove particulate inorganic carbon, washed with distilled water to remove excess acid, dried at 50 °C, and stored in hermetic plastic bottle with silica gel to await organic carbon (CHN) analysis, as described above. Due to the high variability in the diameters of the FPa used for elemental carbon analysis in the time series data from 1995 and 2004 (Fig. 2), average POCs of 0.26 mg mm^−3^ were used for small FPa (<100 µm diameter) and 0.041 mg mm^−3^ for large FPa (>100 µm diameter).

### Total carbonate, coccolith abundance, and coccolithophore composition in FPa

110 intact FPa (50–340 μm in diameter) were removed from sediment trap samples deployed at CQ (44 FPa pooled in 16 bunches) and CC (66 FPa pooled in 18 bunches) throughout the study period. This was made with the aid of a stereomicroscope (Leica model MZ6) at ×4 magnification and placed in a Petri dish with pre-filtered de-carbonated mineral water to prevent coccolith dissolution. Faecal pellets were washed five to eight times with filtered de-carbonated water and photographed. Images were processed with ImageJ software to estimate faecal pellet size and biovolume (Figs 2, 4) by applying prolate spheroid geometry^66^.

In order to analyse the composition of coccospheres and coccoliths within FPa, 1–10 pellets (number depending on size) were removed from the Petri dish, gently placed in 2.5-mL vials containing 2 mL de-carbonated mineral water and sonicated at 50 to 60 Hz for 30 seconds^67^. The contents of the vials were placed in sedimentation chambers and coccospheres and coccoliths identified and counted with an inverted microscope (×1,000 magnification). The coccoliths and coccospheres observed in a known area of the bottom of the chamber were counted and the data extrapolated to the total volume of the faecal pellets^6^. The samples also were examined by scanning electron microscopy to confirm the coccolithophores species identified with light microscopy. Finally, the number of coccoliths per coccolithophore species were transformed to CaCO_3_ using conversion factors from the literature^68^, and the result was extrapolated to the total biovolume of the FPa measured for each sample.

### Statistical analyses

A Kolmogorov-Smirnov test (Minitab software) showed that our data for POC and CaCO_3_ fluxes mediated by FPa in the CQ and CC sites were not distributed normally. We therefore used the non-parametric Mann Whitney test for multiple statistical comparisons (confidence limits set at 95%).

## Supplementary information

Carbon and calcium carbonate export driven by appendicularian faecal pellets in the humboldt current system off chile
