# Supplementary material for: Carbon and Calcium Carbonate Export Driven by Appendicularian Faecal Pellets in the Humboldt Current System off Chile
Source: Sci Rep. 2019 Nov 11;9:16501. doi: 10.1038/s41598-019-52469-y (PMC6848121; doi:10.1038/s41598-019-52469-y)
Supplement: Supplementary file 1 — Carbon and calcium carbonate export driven by appendicularian faecal pellets in the humboldt current system off chile [file 41598_2019_52469_MOESM1_ESM.docx]

**CARBON AND CALCIUM CARBONATE EXPORT DRIVEN BY APPENDICULARIAN FAECAL PELLETS IN THE HUMBOLDT CURRENT SYSTEM OFF CHILE**

Eduardo Menschel A. ^1^ & Humberto E. González ^2^

^1^ Programa Doctorado en Oceanografía de la Universidad de Concepción and Instituto de Ciencias Marinas y Limnológicas, Universidad Austral de Chile, Casilla 567 Valdivia, Chile and Research Center on Dynamics of High Latitude Marine Ecosystems (FONDAP-IDEAL).

^2^ Instituto de Ciencias Marinas y Limnológicas, Universidad Austral de Chile, Casilla 567, Valdivia, Chile and Research Center on Dynamics of High Latitude Marine Ecosystems (FONDAP-IDEAL).

|  |  |  |  |
| --- | --- | --- | --- |
| Taxa | Species | Faeces/animal/day | Reference |
| Copepods | *Acartia tonsa* | 1-75 | Reeve & Walter (1977)^69^; Honjo & Roman (1978)^70^; Petipa (1980)^71^; Gaudy (1974)^72^ |
|  | *Acartia clausi* | 24.5-90.7 | Honjo & Roman (1978)^70^ |
|  | *Calanus finmarchicus* | 0.2-102 | Marshall & Orr (1955a,b)^73-74^; Raymont & Gross (1942)^75^; Bathmann et al. (1990b)^76^ |
|  | *Calanus helgolandicus* | 0.3-250 | Corner et al. (1972, 1974, 1986)^77-79^; Gaudy (1974)^72^; Volkman et al. (1980)^80^ |
|  | *Centropages ponticus* | 8-80 | Petipa (1980)^71^ |
|  | *Centropages typicus* | 0.5-33 | Gaudy (1974)^72^ |
|  | *Eucalanus piteatus* | 55-160 | Paffenhöfer & Knowles (1979)^81^ |
|  | *Oithona minuta* | 5-11 | Petipa (1980)^71^ |
|  | *Pseudocalanus elongatus* | 2-75 | Urry (1965)^82^ |
|  | *Temora stylifera* | 2-72 | Gaudy (1974)^72^; Abou Debs (1984)^83^ |
|  | *Temora turbinata* | 8-169 | Paffenhöfer & Knowles (1979)^81^ |
|  |  |  |  |
| Euphausiids | *Euphausia superba* | 4-40 | Antezana et al. (1982)^84^ |
|  |  |  |  |
| Thaliaceans | *Pyrosoma atlanticus* | 338-1729 | Drits et al. (1992)^85^ |
|  |  |  |  |
| Appendicularians | *Oikopleura dioica* | 25-194 | Vargas et al. (2002)^27^; Bedo et al. (1993)^86^ |
|  | *Oikopleura longicauda* | 243 | Taguchi (1982)^57^ |
|  |  |  |  |

Supplementary Table S1. Functional groups of zooplankton and their faecal pellet production from different regions in the world oceans.

|  |  |  |  |  |  |  |  |  |
| --- | --- | --- | --- | --- | --- | --- | --- | --- |
| Type of faecal pellet | | Vertical flux | | Depth | Reference | | | |
|  | | (mg C m^-2^ d^-1^) | | (m) |  | | | |
| Copepods/Euphausids | | 48 | | 100-300 | González et al. (2000)^15^ | | | |
|  |  | 1-54 | | 20-90 | Wexels et al. (2007)^87^ | | | |
| Copepods | | 0.04-0.6 | | 170 | Gleiber et al. (2012)^88^ | | | |
|  |  | 18,4 | | 100 | Bathmann et al. (1987)^55^ | | | |
| Euphausids | | 0.43 | | 2,300 | Menschel & González (unpublished data) | | | |
|  |  | 0.14-11.4 | | 170 | Gleiber et al. (2012)^88^ | | | |
| Appendicularians | | 0.86-1.4 | | 2,300 | This study | | | |
|  |  | 4.3-21.7 | | 50-100 | González et al (1994)^32^ | | | |
|  |  | 0.05-3.1 | | 500-300 | Wilson et al. (2008)^89^ | | | |
|  |  | 0.57 | | 2,300 | Menschel & González (unpublished data) | | | |
| Salps | | 0.8-23.4 | | <500 | Phillips et al. (2009)^90^ | | | |
| Chaetognaths | | 0.7-41.8 | | 360 | Wefer & Fischer (1991)^91^ | | | |
|  |  |  |  |  |  |  |  |  |

Supplementary Table S2. Vertical carbon flux of different zooplankton taxa estimated in different regions of the world oceans.

|  |  |  |  |  |  |  |
| --- | --- | --- | --- | --- | --- | --- |
| Taxa | Sinking rate | | Reference | | | |
|  | (m d^-1^) | |  | | | |
| Copepods | 5-220 | | Smayda (1971)^92^; Turner (1977)^93^; Honjo & Roman (1978)^70^;  Paffenhöfer & Knowles (1979)^81^; Small et al. (1979)^94^; Bienfang (1980)^95^; Yoon et al., (2001)^96^. | | | |
| Euphausiids | 16-862 | | Fowler & Small (1972)^97^; Youngbluth et al. (1989)^98^; Yoon et al. (2001)^96^ | | | |
| Doliolids | 41-504 | | Bruland & Silver (1981)^99^; Deibel (1990)^100^ | | | |
| Appendicularians | 25-166 | | Gorsky et al. (1984)^101^ | | | |
| Chaetognaths | 27-1,313 | | Dilling & Alldredge (1993)^102^; Giesecke et al., (2010)^103^ | | | |
| Pteropods | 120-1,800 | | Bruland & Silver (1981)^99^; Yoon et al., (2001)^96^ | | | |
| Heteropods | 120-646 | | Yoon et al. (2001)^96^ | | | |
| Salps | 43-368 | | Madin (1982)^104^; Yoon et al. (2001)^96^ | | | |
|  |  |  |  |  |  |  |

Supplementary Table S3. Sinking rate of fecal pellets from different taxa of zooplankton taxa reported in the literature.

| **FPa production rate and abundance** | Average | References / Sources | |
| --- | --- | --- | --- |
| 1) Faeces Production FPa h^-1^: 8.1; 7.3; 7.6; 10.1 | 8.28 FPa h^-1^ (198.6 FPa d^-1^) (72,500 FPa y^-1^) | 27,86,57 | |
| 2) Abundance of appendicularians m^-3^ | (6.0; 3.3; 0.2; 5.2; 132.1; 3.4; 0.8 m^-3^): Average 21.6 | [www.st.nmfs.noaa.gov/copepod/atlas](http://www.st.nmfs.noaa.gov/copepod/atlas); 49 | |
| 3) Appendicularian abundance | 1,078 m^-2^; 1.08 x 10^9^ km^-2^ | Integrated within the upper 50 m | |
| **FPa numbers, volumes and carbon content** | | | |
| 4) PFa (Number km^-2^ y^-1^) | 7.8 x 10^13^ | Within the upper 50 m water column | |
| 5) Area HCS (18.5-42°S) (2600 x 350 km) | 910.000 km^2^ |  | |
| 6) PFa in the whole HCS y^-1^ | 7.1 x 10^19^ | Integrated within the upper 50 m | |
| 7) Small (<100 µm diameter) FPa volume | 0.0014 mm^3^ | This contribution | |
| 8) Small FPa C mm^-3^ | 0.26 mgC mm^-3^ | This contribution | |
| 9) Small FPa carbon content | 0.000364 mgC |  | |
| 10) Large (>100 µm diameter) FPa volume | 0.0364 mm^3^ | This contribution | |
| 11) Large FPa C mm^-3^ | 0.041 mgC mm^-3^ | This contribution | |
| 12) Large FPa carbon content | 0.0015 mgC |  | |
| 13) Small FPa (90% of total in the HCS) | 6.4 x 10^19^ |  | |
| 14) Small FPa carbon content in the HCS | 2.33 x 10^16^ mgC y^-1^ | Integrated | |
| 15) Large FPa (10% of total in the HCS) | 7.1 x 10^18^ | Integrated | |
| 16) Large FPa carbon content in the HCS | 1.07 x 10^16^ mgC y^-1^ | integrated | |
| 17) Total FPa carbon content in the upper | 3.4 x 10^16^ mgC y^-1^ | 3.4 x 10^7^ tonC y^-1^; 0.034 GtC y^-1^ | |
| **FPa export at CC and CQ sites** |  |  | |
| 18) FPa export (CQ and CC) | 1.04 mgC m^-2^ d^-1^ | 379.6 kgC km^-2^ y^-1^ | |
| 19) FPa export (in the HCS) | 345.436.000 kgC km^-2^ y^-1^ | 0.000345 GtC HCS^-1^ y^-1^ | |
| 20) FPa export efficiency towards 2,300 m depth | 1% |  | |
|  |  |  | |
|  |  |  |  |

Supplementary Table S4. Summary of data used for calculations of FPa carbon flux at 2,300 m in both study area (CQ and CC) and for the Humboldt Current System of Chile of Chile between 18.5 and 41.5°S.

|  |  |  |  |  |  |  |  |  |
| --- | --- | --- | --- | --- | --- | --- | --- | --- |
| Site | No. of Pellet | length FPa (mean in mm) | width FPa (mean in mm) | BioVol Total mm^3^ | Volume (mean mm^3)^ | SD mm^3^ | mgC total | mgC mm3 |
| CC | 51 | 0,199 | 0,083 | 0,039 | 0,001 | 0,0003 | 0,024 | 0,624 |
| CC | 28 | 0,458 | 0,176 | 0,224 | 0,007 | 0,0042 | 0,029 | 0,127 |
| CC | 14 | 0,930 | 0,340 | 0,826 | 0,056 | 0,0286 | 0,033 | 0,040 |
| CC | 49 | 0,229 | 0,089 | 0,049 | 0,001 | 0,0003 | 0,031 | 0,639 |
| CC | 115 | 0,224 | 0,089 | 0,115 | 0,001 | 0,0005 | 0,026 | 0,225 |
| CC | 171 | 0,175 | 0,069 | 0,083 | 0,000 | 0,0003 | 0,041 | 0,500 |
| CC | 142 | 0,242 | 0,099 | 0,190 | 0,001 | 0,0007 | 0,035 | 0,185 |
| CC | 16 | 0,941 | 0,353 | 1,040 | 0,061 | 0,0330 | 0,053 | 0,051 |
| CQ | 36 | 0,548 | 0,182 | 0,361 | 0,010 | 0,0043 | 0,042 | 0,117 |
| CQ | 18 | 0,878 | 0,315 | 0,907 | 0,046 | 0,0293 | 0,038 | 0,042 |
| CQ | 44 | 0,539 | 0,187 | 0,455 | 0,010 | 0,0044 | 0,063 | 0,139 |
| CQ | 35 | 0,603 | 0,216 | 0,537 | 0,015 | 0,0057 | 0,069 | 0,128 |
| CQ | 18 | 0,900 | 0,318 | 0,919 | 0,048 | 0,0266 | 0,044 | 0,048 |
|  |  |  |  |  |  |  |  |  |

Supplementary Table S5. Detail of the PFa measured for the CHN analysis for the CC and CQ sites.

**References**

69. Reeve, M. R. & Walter, M. A. Observations on the existence of lower threshold and upper critical food concentrations for the copepod *Acartia tonsa* Dana. *J. Exp. Mar. Biol. Ecol*. **29**, 211-221 (1977).

70. Honjo, S. & Roman, M. R. Marine copepod fecal pellets: production, preservation and sedimentation. *J. Mar. Res*. **36**, 45-57 (1978).

71. Petipa, T. S. Food interrelationship as the basis of matter and energy turnover in marine ecosystems, Production primaire et secondaire, colloquies franco-sovietique, *Stn. Mar. Endoume* 9-12 janvier 1979. Publ. C.N.E.X.O. **10**, 51-62 (1980).

72. Gaudy, R. Feeding of four species of pelagic copepods under experimental conditions. *Mar. Biol*. **25**, 125-141 (1974).

73. Marshall, S. M. & Orr, A. P. On the biology of *Calanus finmarchicus*. 8. Food uptake, assimilation and excretion in adult and stage V *Calanus*. *J. Mar. Biol*. Ass. U. K. **34**, 495-529 (1955a).

74. Marshall, S. M. & Orr, A. P. Experimental feeding of the copepod *Calanus finmarchicus* (Gunner) on phytoplankton cultures labelled with radioactive carbon (14C). *Deep-Sea Res*. **3**, 110-114 (1955b).

75. Raymont, J. E. G. & Gross, F. On the feeding and breeding of *Calanus finmarchicus* under laboratory conditions. *Proc. Royal Soc*. Edinburgh LXI, III **20**, 267-287 (1942).

76. Bathmann, U. V., Noji, T. T. & von Bodungen B. Copepod grazing potential in late winter in the Norwegian Sea - a factor in the control of spring phytoplankton growth?. *Mar. Ecol. Prog. Ser*. **60**, 225-233 (1990b).

77. Corner, E. D. S., Head, R. N. & Kilvington, C. C. On the nutrition and metabolism of zooplankton. VIII. The grazing of Biddulphia cells by *Calanus helgolandicus*. *J. Mar. Biol*. *Ass. U. K.* **52**, 847-861 (1972).

78. Corner, E. D. S., Head, R. N. & Kilvington, C. C. On the nutrition and metabolism of zooplankton. IX. Studies relating to the nutrition of overwintering *Calanus.* *J. Mar. Biol*. *Ass. U. K.* **54**, 319-331 (1974).

79. Corner, E. D. S., O’Hara, C. M., Neal, A. C. & Eglinton, G. The biological chemistry of marine copepods. (eds. Corner E. D. & O’ Hara S. C. m) 260-321 (Clarendon Press, Oxford, 1986).

80. Volkman, J. K., Corner, D. S. Eglinton, G. Colloq. int. CNRS n° 293 (ed. Daumas, R.) 185-197 (Editions CNRS, Paris, 1980).

81. Paffenhöfer, G-A. & Knowles, S. C. Ecological implications of faecal pellets size, production and consumption by copepods. *J. Mar. Res*. **37**, 35-49 (1979).

82. Urry, D. L. Observations on the relationship between the food and survival of *Pseudocalanus elongatus* in the laboratory. *J. Mar. Biol*. *Ass. U. K.* **45**, 49-58 (1965).

83. Abou Debs, C. Carbon and nitrogen budget of the calanoid copepod *Temora stylifera*: effect of concentration and composition of food. *Mar. Ecol. Prog. Ser*. **15**, 213-223 (1984).

84. Antezana, T., Ray, K. & Melo, C. Trophic behavior of *Euphausia superba* Dana in laboratory conditions. *Polar Biol*. **1**, 77-82 (1982).

85. Drits, A.V., Arashkevich, E. G. & Semenova, T. N. *Pyrosoma atlanticus* (Tunicata, Thaliacea): grazing impact on phytoplankton standing stock and role in organic carbon flux. *J. Plankton Res*. **14**, 799-809 (1992).

86. Bedo, A. W., Acuña, J. L. Robins, D. & Harris, R. P. Grazing in the micron and the sub-micron particle size range: The case of *Oikopleura dioica* (Appendicularia). *Bull. Mar. Sci*. **53**(1), 2-14 (1993).

87. Wexels, R. C., Reigstad, M., Wassmann, P., Arashkevich, E. & Falk-Petersen, S. Export or retention? Copepod abundance, faecal pellet production and vertical flux in the marginal ice zone through snap shots from the northern Barents Sea. *Polar Biol*. **30**, 719-730 (2007).

88. Gleiber, M. R., Steinberg, D. K & Ducklow, H. W. Time series of vertical flux of zooplankton fecal pellets on the continental shelf of the western Antarctic Peninsula. *Mar. Ecol. Prog. Ser*. **471**, 23-36 (2012).

89. Wilson, S. E., Steinberg, D. K. & Buesseler, K. O. Changes in fecal pellet characteristics with depth as indicators of zooplankton repackaging of particles in the mesopelagic zone of the subtropical and subarctic North Pacific Ocean*. Deep-Sea Res*. II. **55**, 1636-1647 (2008).

90. Phillips, B., Kremer, P. & Madin, L. P. Defecation by *Salpa thompsoni* and its contribution to vertical flux in the Southern Ocean. *Mar. Biol*. **156**, 455-467 (2009).

91. Wefer, G. & Fischer, G. Annual primary production and export ﬂux in the Southern Ocean from sediment trap data. *Mar. Chem*. **35**, 597-613 (1991).

92. Smayda, T. J. Normal and accelerated sinking of phytoplankton in the sea. *Mar. Geol*. **11**, 105-122 (1971).

93. Turner, J. T. Sinking rates of fecal pellets from the marine copepod *Pontella meadii*. *Mar. Biol*. **40**, 249-259 (1977).

94. Small, L. F., Fowler, S. W. & Ünlü, M. I. Sinking rates of natural copepod fecal pellets. *Mar. Biol*. **51**, 233-241 (1979).

95. Bienfang, P. K. Herbivore diet affects fecal pellet settling. *Can. J. Fish. Aquat. Sci*. **37**, 1352-1357 (1980).

96. Yoon, W. D., Kim, S. K. & Han, K. N. Morphology and sinking velocities of fecal pellets of copepod, molluscan, euphausiid and salp taxa in the northeastern tropical Atlantic. *Mar. Biol*. **139**, 923-928 (2001).

97. Fowler, S. W. & Small, L. F. Sinking rates of euphausiid faecal pellets. *Limnol. Oceanogr*. **17**, 293-296 (1972).

98. Youngbluth, M. J., Bailey, T. G., Davoll, P. J., Jacoby, C. A., Blades-Eckelbarger, P. I. *et al*. Fecal pellet production and diel migratory behavior by the euphausiid *Meganyctiphanes norvegica* effect benthic-pelagic coupling. *Deep-Sea Res*. **36**, 1491-1501 (1989).

99. Bruland, K. W. & Silver, M. W. Sinking rates of fecal pellets from gelatinous zooplankton (salps, pteropods, doliolids). *Mar. Biol*. **63**, 295-300 (1981).

100. Deibel, D. Still-water sinking velocity of fecal material from the pelagic tunicate *Dolioletta gegenbauri*. *Mar. Ecol. Prog. Ser*. **62**, 55-60 (1990).

101. Gorsky, G., Palazzoli, I. & Fenaux, R. Premières données sur la respiration des appendiculaires (tuniciers pélagiques). *CR Hebd Seances Acad Sci Paris*. **298**, 531- 534 (1984).

102. Dilling, L. & Alldredge, A. L. Can chaetognath fecal pellets contribute significantly to carbon flux? *Mar. Ecol. Prog. Ser*. **92**, 51-58 (1993).

103. Giesecke, R., González, H. E. & Bathmann, Ulrich. The role of the chaetognath *Sagitta gazellae* in the vertical carbon ﬂux of the Southern Ocean. *Polar Biol*. **33**, 293-304 (2010).

104. Madin L. P. Production, composition and sedimentation of salp fecal pellets in oceanic water. *Mar. Biol***. 67**, 39-45 (1982).
